# Humoral hypercalcemia of pregnancy treated with bisphosphonates

**DOI:** 10.20945/2359-3997000000016

**Published:** 2018-01-01

**Authors:** Ronit Koren, Ortal Neeman, Shlomit Koren, Carlos A. Benbassat

**Affiliations:** 1 Assaf Harofeh Medical Center Department of Internal Medicine A Zerifin Israel Department of Internal Medicine A, Assaf Harofeh Medical Center, Zerifin, Israel; 2 Assaf Harofeh Medical Center Department of Obstetrics and Gynecology Division of Maternal Fetal Medicine Zerifin Israel Division of Maternal Fetal Medicine, Department of Obstetrics and Gynecology, Assaf Harofeh Medical Center, Zerifin, Israel; 3 Tel-Aviv University Sackler Faculty of Medicine Tel-Aviv Israel Sackler Faculty of Medicine, Tel-Aviv University, Tel-Aviv, Israel; 4 Assaf Harofeh Medical Center Endocrine Institute Zerifin Israel Endocrine Institute, Assaf Harofeh Medical Center, Zerifin, Israel; 5 Assaf Harofeh Medical Center Diabetes Unit Zerifin Israel Diabetes Unit, Assaf Harofeh Medical Center, Zerifin, Israel

## Abstract

Hypercalcemia can be hazardous during pregnancy, most cases being due to primary hyperparathyroidism. We report a case of hypercalcemia with suppressed PTH levels necessitating treatment with bisphosphonates during pregnancy. A 38-year-old woman at the 26^th^ week gestation was admitted because of symptomatic hypercalcemia. She did not take any medication that could influence her calcium levels. Physical examination was unremarkable. Laboratory tests on admission were: calcium 12.7 mg/dL (8.5-10.5 mg/dL), phosphorus 1.8 mg/dL (2.5-4.5 mg/dL) and PTH on 3 consecutive tests 1.2, 1.3 and 1.2 pg/mL (15-65 pg/mL). Her 24h urine calcium was 900 mg, 25-OH-D 40 ng/mL (30-58 ng/mL) and 1,25-OH-D 99 pg/mL (80-146 for women in the third trimester). Abdominal ultrasound revealed multiple hypervascular liver lesions consistent with hemangiomas by MRI. Breast and neck ultrasound were normal, and chest CT revealed few non-significant 0.3-0.7 cm pulmonary nodules with no change after an interval of 3 months. She was treated with isotonic saline, loop diuretics and calcitonin. Despite this treatment, calcium levels remained high (14.1 mg/dL), and pamidronate was initiated. On 35^th^ week gestation, she underwent a cesarean section complicated by hypocalcemia of the newborn. Eight weeks after delivery, her calcium levels are 9.4 mg/dL and PTH 18 mg/dL. According to the extensive workup and the post-partum normalization of PTH and calcium levels, we conclude that excessive secretion of placental PTHrP was the cause of hypercalcemia in this patient. No significant adverse effect of bisphosphonate on the mother or baby were seen at the short term follow up.

## INTRODUCTION

Hypercalcemia can be hazardous during pregnancy, leading to maternal and fetal complications. Hypercalcemia is challenging to diagnose during this period because physiological changes such as hemodilusion, hypoalbuminemia, an elevated glomerular filtration rate, hypercalciuria and placental transfer of calcium to the fetus may all lead to lower blood calcium levels. Most cases of hypercalcemia diagnosed during pregnancy are due to primary hyperparathyroidism (PHPT) caused by a solitary adenoma. Less commonly, hypercalcemia is caused by multiple parathyroid adenomas, diffuse hyperplasia and parathyroid carcinoma (1). When maternal PTH levels are suppressed, the diagnostic challenge is further increased. Several cases have been reported of women with hypercalcemia secondary to milk alkali syndrome (2,3), and cases of parathyroid-related-protein (PTHrP) mediated hypercalcemia have been described during pregnancy in women with uterine leiomyoma (4), neuroendocrine tumor of the pancreas (5) and ovarian clear cell carcinoma (6). Rare cases of humoral hypercalcemia of pregnancy, in which the PTHrP source was suspected to be the placenta, have also been described previously in the literature (7,8).

It is probable that most cases of hypercalcemia due to PHPT go unrecognized and are not associated with maternal or fetal complications (9). When symptomatic, women can present with nausea, vomiting, confusion, agitation, nephrolithiasis, pancreatitis, hyperemesis gravidarum and preeclampsia. Hypercalcemic crisis has been reported with calcium levels above 14 mg/dL leading to uremia, coma and maternal death. The most serious fetal complications involve suppressed parathyroid gland with severe hypocalcemia, tetany and fetal or neonatal demise (10-13).

We report a case of hypercalcemia with suppressed parathyroid hormone (PTH) levels necessitating treatment with bisphosphonates during pregnancy.

## CASE REPORT

A 38-year-old woman at the 26^th^ week gestation was admitted to hospital because of elevated calcium levels. Her first pregnancy 16 years prior to presentation was uneventful. She was a carrier of the hemophilia gene and was generally healthy. Her symptoms consisted of pruritus, polidypsia and polyuria. She did not take any medication or food supplement that could influence her calcium levels. Physical examination was unremarkable. Laboratory tests were as follows: calcium 12.7 mg/dL (reference range, 8.5-10.5 mg/dL), albumin 3.6 mg/dL (3.5-5.2 g/dL), ionized calcium 7.9 mg/dL (4-4.9), magnesium 1.21 (1.8-2.6 mg/dL), phosphorus 1.8 mg/dL (2.5-4.5 mg/dL) and PTH levels on 3 consecutive tests 1.2, 1.3 and 1.2 pg/mL (15-65 pg/mL). Her 24h urine calcium was 900 mg, 25-hydroxyvitamin (25OH) D- 40 ng/mL (30-58 ng/mL), 1,25OH vitamin D- 99 pg/mL (16-80 pg/mL for the general population, 80-146 for women in the third trimester (14). Thyroid function tests and angiotensin-converting enzyme levels were normal. Further evaluation included abdominal ultrasound, which revealed multiple hypervascular liver lesions that were later diagnosed as liver hemangiomas by MRI; breast and neck ultrasounds, which were normal; and chest CT, which revealed small pulmonary nodules (0.3-0.7 cm) that had not changed at a follow up study 3 months afterward. She was treated with isotonic saline (up to 250 mL/hour) and loop diuretics (40 mg/day) and received calcitonin (300 units twice a day, than 200 units twice daily for 2 days). Urinary output reached 13.5 liter/day. Since calcium levels rose up to 14 mg/dL (corrected to albumin) after this vigourus therapy, treatment with pamidronate was initiated. A total of 90 mg was given in three divided doses with a good response. At the 35^th^ week gestation, after calcium levels began to rise again, induction of delivery was ensued. Due to no progression, she underwent a cesarean section complicated by hypocalcemia of the newborn. Eight weeks after delivery, her calcium levels were 9.4 mg/dL, phosphorus 3.4, 1,25 OH vitamin D 49 mg/dL and PTH 18 pg/mL. Four months after delivery, maternal and infant's calcium levels were within the normal range, and the infant's height and weight growth were adequate.

## DISCUSSION

Regulation of calcium and phosphorus during fetal life is of utmost importance for proper bone development and mineralization. This regulation is dependent on maternal PTH and PTHrP (15). During pregnancy, maternal intestinal absorption of calcium more than doubles as a response to the increasing demand. Factors responsible for this are calcitriol and placental lactogen among other things. Several changes become apparent more obviously during the 3^rd^ trimester and during lactation: free calcitriol levels rise (16), maternal bone resorption becomes evident (due to an increase in urine cross-linked N-telopeptides of type I collagen, especially during the winter) (17), and PTHrP levels, which rise steadily during pregnancy, undergo further elevation during lactation (18).

PTHrP can be secreted physiologically from the lactating breast, placenta, pregnant uterus and various benign and malignant tumors. It has an effect on chondrocyte differentiation and an anabolic function on bone. PTHrP signaling is required for the formation of the mammary glands and is related to calcium transfer across the placenta (19). PTHrP can reach the circulation and cause hypercalcemia (7,20). Its role in maternal bone resorption and osteoporosis of pregnancy and lactation has not been elucidated yet (16,21).

After the extensive workup, and in view of the normalization of calcium levels after delivery, it is our opinion that the cause of the presented patient's hypercalcemia was overproduction of PTHrP in the placenta. It is possible that PTHrP was secreted from mammary glands, or from another unrevealed source. Another possibility is that this is a case of aberrant calcium homeostasis not related to pregnancy. Unfortunately, PTHrP measures are unavailable to us.

Calcitriol levels rise during normal pregnancy. PTH is normally the dominant regulator of Cyp27b1 in adults. During pregnancy the marked increase in calcitriol occurs while PTH is often suppressed to low levels, which suggests that PTH is not responsible for the upregulation of Cyp27b1 (22). Reference values for pregnancy have been suggested and are significantly higher than for the general population and even higher during the third trimester (14).

It is suggested that estradiol, prolactin and placental lactogen, which are elevated during pregnancy, may in part stimulate Cyp27b1, as suggested by animal data. Although the placenta was previously considered to be the source of calcitriol during pregnancy, it is nowadays believed that it is mainly secreted from the maternal kidney (23). In the present case calcitriol levels were not elevated when compared to pregnancy reference values, and hence, it is our view that PTHrP might be a reasonable explanation for the hypercalcemia presented in this case. Other pregnancy-related hormones working excessively or aberrantly might have been the culprit, but the limited laboratory possibilities prevented us from reaching the final conclusion.

Bisphosphonates are synthetic analogues of pyrophosphate that inhibit bone resorption. They are absorbed into the mineral surface of the bone where they interfere with the action of osteoclasts (24). The slow release of bisphosphonates from the bone causes detectable levels in urine for many weeks and months after discontinuing the drug (25).

Bisphosphonates are not considered an accepted treatment during pregnancy, as animal studies found adverse effects on the fetus's skeleton. In rats, bisphosphonates were found to cross the placenta, accumulate in the skeleton of the fetus and decrease fetal weight and bone growth (26). It was also shown to cause symptomatic hypocalcemia of the dams and even fetal demise (27). It is noted that doses administered in animal studies were much higher than those used in clinical practice.

Data regarding the effects of bisphosphonates on human reproduction are scarce. Case reports of inadvertent exposure during pregnancy or pre-pregnancy administration had no apparent adverse effect on the embryo or fetus, although the newborn can develop hypocalcemia in the first few days of life (28). In a review of 65 maternal-fetal pairs with a wide variety of agents and doses, bisphosphonates were related to a small decrease in gestational age and birthweight and hyper or hypocalcemia of the newborn. No long-term maternal or neonatal adverse effects were reported (29). In this case, decision making was established by a multidisciplinary team constituted by endocrinologists, gynecologists, a nephrologist and a clinical pharmacologist. As calcium levels were elevated and tended to further increase despite the reception of accepted treatment, it seemed prudent to make another, more significant intervention even though the data was not sufficient.

In conclusion, hypercalcemia during pregnancy constitutes a diagnostic and management challenge. We add our experience in evaluating hypercalcemia with decreased PTH levels and the use of bisphosphonates during pregnancy with good results.

## References

[1] 1. Dochez V, Ducarme G. Primary hyperparathyroidism during pregnancy. Arch Gynecol Obstet. 2015;291(2):259-63. 10.1007/s00404-014-3526-8 25367603

[2] 2. Kleinman GE, Rodriquez H, Good MC, Caudle MR. Hypercalcemic crisis in pregnancy associated with excessive ingestion of calcium carbonate antacid (milk-alkali syndrome): successful treatment with hemodialysis. Obstet Gynecol. 1991;78(3 Pt 2):496-9. 1870805

[3] 3. Gordon MV, McMahon LP, Hamblin PS. Life-threatening milkalkali syndrome resulting from antacid ingestion during pregnancy. Med J Aust. 2005;182(7):350-1. 15804228

[4] 4. Rahil A, Khan FY. Humoral hypercalcemic crisis in a pregnant woman with uterine leiomyoma. J Emerg Trauma Shock. 2012;5(1):87-9. 10.4103/0974-2700.93093 PMC3299164 22416164

[5] 5. Ghazi AA, Boustani I, Amouzegar A, Attarian H, Pourafkari M, Gashti HN, et al. Postpartum hypercalcemia secondary to a neuroendocrine tumor of pancreas; a case report and review of literature. Iran J Med Sci. 2011;36(3):217-21. PMC3556773 23358529

[6] 6. Hwang CS, Park SY, Yu SH, Park JY, Park CT, Han KO. Hypercalcemia induced by ovarian clear cell carcinoma producing all transcriptional variants of parathyroid hormone-related peptide gene during pregnancy. Gynecol Oncol. 2006 Nov;103(2):740-4. 10.1016/j.ygyno.2006.05.051 16956653

[7] 7. Lepre F, Grill V, Ho PW, Martin TJ. Hypercalcemia in pregnancy and lactation associated with parathyroid hormone-related protein. N Engl J Med. 1993;328(9):666-7. 10.1056/NEJM199303043280917 8429868

[8] 8. Sato K. Hypercalcemia during pregnancy, puerperium, and lactation: review and a case report of hypercalcemic crisis after delivery due to excessive production of PTH-related protein (PTHrP) without malignancy (humoral hypercalcemia of pregnancy). Endocr J. 2008;55(6):959-66. 10.1507/endocrj.k08e-092 18614854

[9] 9. Hirsch D, Kopel V, Nadler V, Levy S, Toledano Y, Tsvetov G. Pregnancy outcomes in women with primary hyperparathyroidism. J Clin Endocrinol Metab. 2015;100(5):2115-22. 10.1210/jc.2015-1110 25751112

[10] 10. Truong MT, Lalakea ML, Robbins P, Friduss M. Primary hyperparathyroidism in pregnancy: a case series and review. Laryngoscope. 2008;118(11):1966-9. 10.1097/MLG.0b013e318180276f 18758377

[11] 11. Lee CC, Chao AS, Chang YL, Peng HH, Wang TH, Chao A. Acute pancreatitis secondary to primary hyperparathyroidism in a postpartum patient: a case report and literature review. Taiwan J Obstet Gynecol. 2014;53(2):252-5. 10.1016/j.tjog.2013.01.029 25017280

[12] 12. Gokkaya N, Gungor A, Bilen A, Bilen H, Gviniashvili D, Karadeniz Y. Primary hyperparathyroidism in pregnancy: a case series and literature review. Gynecol Endocrinol. 2016;32(10):783-6. 10.1080/09513590.2016.1188916 27243597

[13] 13. Walker A, Fraile JJ, Hubbard JG. “Parathyroidectomy in pregnancy”-a single centre experience with review of evidence and proposal for treatment algorithim. Gland Surg. 2014;3(3):158-64. 10.3978/j.issn.2227-684X.2014.02.04 PMC4139131 25207208

[14] 14. Souberbielle JC, Cavalier E, Delanaye P, Massart C, Brailly-Tabard S, Cormier C, et al. Serum calcitriol concentrations measured with a new direct automated assay in a large population of adult healthy subjects and in various clinical situations. C Clin Chim Acta. 2015;451(Pt B):149-53. 10.1016/j.cca.2015.09.021 26409159

[15] 15. Kovacs CS. Bone development and mineral homeostasis in the fetus and neonate: roles of the calciotropic and phosphotropic hormones. Physiol Rev. 2014;94(4):1143-218. 10.1152/physrev.00014.2014 25287862

[16] 16. Kovacs CS. Calcium and bone metabolism disorders during pregnancy and lactation. Endocrinol Metab Clin North Am. 2011;40(4):795-826. 10.1016/j.ecl.2011.08.002 22108281

[17] 17. O'Brien EC, Kilbane MT, McKenna MJ, Segurado R, Geraghty AA, McAuliffe FM. Calcium intake in winter pregnancy attenuates impact of vitamin D inadequacy on urine NTX, a marker of bone resorption. Eur J Nutr. 2017 Feb 21. 10.1007/s00394-017-1385-3 28224220

[18] 18. Kovacs CS. Calcium and bone metabolism during pregnancy and lactation. J Mammary Gland Biol Neoplasia. 2005;10(2):105-18. 10.1007/s10911-005-5394-0 16025218

[19] 19. Wysolmerski JJ. Parathyroid hormone-related protein: an update. J Clin Endocrinol Metab. 2012;97(9):2947-56. 10.1210/jc.2012-2142 PMC3431578 22745236

[20] 20. Khosla S, van Heerden JA, Gharib H, Jackson IT, Danks J, Hayman JA, et al. Parathyroid hormone-related protein and hypercalcemia secondary to massive mammary hyperplasia. N Engl J Med. 1990;322(16):1157. 10.1056/NEJM199004193221613 2320085

[21] 21. Anai T, Tomiyasu T, Arima K, Miyakawa I. Pregnancy-associated osteoporosis with elevated levels of circulating parathyroid hormone-related protein: a report of two cases. J Obstet Gynaecol Res. 1999;25(1):63-7. 10.1111/j.1447-0756.1999.tb01124.x 10067016

[22] 22. Kirby BJ, Ma Y, Martin HM, Buckle Favaro KL, Karaplis AC, Kovacs CS. Upregulation of calcitriol during pregnancy and skeletal recovery after lactation do not require parathyroid hormone. J Bone Miner Res. 2013;28(9):1987-2000. 10.1002/jbmr.1925 23505097

[23] 23. Kovacs CS. Maternal Mineral and Bone Metabolism During Pregnancy, Lactation, and Post-Weaning Recovery. Physiol Rev. 2016;96(2):449-547. 10.1152/physrev.00027.2015 26887676

[24] 24. Fleisch HA. Bisphosphonates: preclinical aspects and use in osteoporosis. Ann Med. 1997;29(1):55-62. 10.3109/07853899708998743 9073324

[25] 25. Cremers S, Sparidans R, den HJ, Hamdy N, Vermeij P, Papapoulos S. A pharmacokinetic and pharmacodynamic model for intravenous bisphosphonate (pamidronate) in osteoporosis. Eur J Clin Pharmacol. 2002;57(12):883-90. 10.1007/s00228-001-0411-8 11936708

[26] 26. Patlas N, Golomb G, Yaffe P, Pinto T, Breuer E, Ornoy A. Transplacental effects of bisphosphonates on fetal skeletal ossification and mineralization in rats. Teratology. 1999;60(2):68-73. 10.1002/(SICI)1096-9926(199908)60:2<68::AID-TERA10>3.0.CO;2-H 10440778

[27] 27. Graepel P, Bentley P, Fritz H, Miyamoto M, Slater SR. Reproduction toxicity studies with pamidronate. Arzneimittelforschung. 1992;42(5):654-67. 1530681

[28] 28. Djokanovic N, Klieger-Grossmann C, Koren G. Does treatment with bisphosphonates endanger the human pregnancy? J Obstet Gynaecol Can. 2008;30(12):1146-8. 10.1016/S1701-2163(16)34026-9 19175968

[29] 29. Green SB, Pappas AL. Effects of maternal bisphosphonate use on fetal and neonatal outcomes. Am J Health Syst Pharm. 2014;71(23):2029-36. 10.2146/ajhp140041 25404594

